# Unrealistically pristine air in the Arctic produced by current global scale models

**DOI:** 10.1038/srep26561

**Published:** 2016-05-25

**Authors:** Yousuke Sato, Hiroaki Miura, Hisashi Yashiro, Daisuke Goto, Toshihiko Takemura, Hirofumi Tomita, Teruyuki Nakajima

**Affiliations:** 1 RIKEN Advanced Institute for Computational Science, Kobe, Japan; 2 Department of Earth and Planetary Science, The University of Tokyo, Tokyo, Japan; 3 National Institute for Environmental Studies, Tsukuba, Japan; 4 Research Institute for Applied Mechanics, Kyushu University, Fukuoka, Japan; 5 Earth Observation Research Center, Japan Aerospace Exploration Agency, Tsukuba, Japan

## Abstract

Black carbon aerosol (BCA) in the Arctic has profound impacts on the global climate system through radiation processes. Despite its enormous impacts, current global scale models, powerful tools for estimating overall impact, tend to underestimate the levels of BCA in the Arctic over several seasons. Using a global aerosol transport simulation with a horizontal grid resolution of 3.5 km, we determined that a higher resolution significantly reduced the underestimation of BCA levels in the Arctic, mainly due to an enhancement of the representation of low-pressure and frontal systems. The BCA mass loading in the Arctic simulated with 3.5-km grid resolution was 4.2-times larger than that simulated with coarse (56-km) grid resolution. Our results also indicated that grid convergence had not occurred on both the contrast between the cloud/cloud free areas and the poleward BCA mass flux, despite the use of the 3.5-km grid resolution. These results suggest that a global aerosol transport simulation using kilometre-order or finer grid resolution is required for more accurate estimation of the distribution of pollutants in the Arctic.

Over the last three decades, black carbon aerosol (BCA), primarily derived from the burning of biomass, agricultural waste, and fossil fuels, has been an important research topic due to extensive impacts on the global climate through atmospheric radiation processes^1^,^2^. BCA absorbs sunlight and heats the atmosphere^3^, and when it accumulates on snow-covered surfaces it reduces reflectance^2^. Through these processes, BCA has played a significant role in the warming of the Arctic^2^ and has become a key topic in climate research. The Fifth Assessment Report of the Intergovernmental Panel for Climate Change (IPCC AR5)^1^ reported that there is still considerable uncertainty in radiative forcing (0.3–1.2 W m^−2^), with the most accurate estimation of 0.64 W m^−2^ being about one-third that of carbon dioxide.

To reduce this uncertainty, many studies have coupled global climate models (GCMs) with aerosol transport models. However, the geographical distribution of BCA simulated by the different models displays considerable variation^4^, and almost all of the models underestimate compared with *in situ* measurements in the Arctic (*e.g.*, sites at Alert, Barrow, and Zeppelin^5^) in spring and winter. A recent modelling study indicated that BCA simulated by the models overestimated BCA levels compared with *in situ* measurements in summer, but the variation between the results of each model was large^6^.

Although, recent studies have reported that the underestimation can be reduced by implementing missing of BCA emission in the Arctic^6^,^7^,^8^, it has been commonly accepted that the BCA found in the Arctic has been carried long distances from the mid-latitudes^9^, due to the limited number of BCA emission sources in the region^10^. Accordingly, it is reasonable to assume that current-generation GCMs should accurately resolve the processes responsible for this long-range transport^11^, as a prerequisite for conducting accurate estimations of Arctic BCA levels. Many studies have attempted to understand the uncertainties of the BCA levels in the Arctic by focusing on emission inventories and the microphysical processes of BCA. One study estimated the BCA emission from Asia and Europe using a data assimilation system (Kalman filter)^12^ and concluded that an emission inventory with a fine resolution could be a solution to reduce the underestimation^8^.

As well as the emission inventory, microphysical processes of BCA have also been investigated in numerous studies. Among several microphysical processes, the aging process has been of particular interest^13^,^14^,^15^. A model study indicated that slow aging resulted in a several-fold larger amount of BCA in the Arctic^14^; a sophisticated aging process scheme was developed for reducing the uncertainties relating to the process^15^. Wet deposition is also a key process in determining the amount of BCA transported^13^. Several studies have indicated that the seasonal cycle of BCA in the Arctic is mainly controlled by the seasonal variability of wet deposition^16^,^17^,^18^; the underestimation of BCA can be reduced by changing the wet deposition scheme^19^. In addition to these two processes, the effects of dry deposition on BCA transport to the Arctic have also been investigated^19^. Despite these efforts to improve the models, the underestimation of the Arctic BCA levels in spring and winter in current-generation GCMs has yet to be resolved^20^.

In addition to microphysical processes, the transport of BCA by mid-latitude low-pressure systems (henceforth, “lows”) that usually accompany warm and cold fronts needs to be addressed in more detail as BCA emitted from continental areas in the Northern Hemisphere (*i.e.*, Europe, East Asia, and North America) is carried poleward through the polar front^21^,^22^. In the region of the polar front, lows with frontal systems, whose lifetime is from one week to two weeks, are frequently generated, and poleward mass transport is continuously induced. However, the detailed structure of the lows, in particular in the vicinity of the fronts (*i.e.*, fine vortex filaments, quasi-discontinuous transition of warm/cold temperatures, and large wind shear), cannot be resolved by current-generation GCMs. This is because the horizontal spatial scale of these frontal systems (several tens of kilometers^23^) is considerably finer than the spatial resolution (several tens to several hundreds of kilometers^6^) of current-generation GCMs.

Several studies have investigated the sensitivity of grid resolution to BCA transport using a regional^14^,^24^ and global scale models^25^,^26^. These studies have suggested that wet deposition is highly dependent on the sub-grid scale cloud fraction. The detailed structure of clouds simulated with a fine grid resolution leads to the reduction of aerosol-cloud interactions, and the reduction enhances BCA transport to the Arctic. However, the finest resolution of these experiments (10 km for regional models, and 0.25° for global models) is still too coarse to reproduce the detailed structure of frontal systems accompanying lows^27^. In addition, the regional models have a limited domain size, and it is difficult to completely remove the effects of the discontinuity near the lateral boundary between the outer model (parent model) and inner model (child model).

A global aerosol transport model with a fine grid resolution (kilometre-order) allows the structure of lows and frontal systems to be resolved for investigation of the effect of grid resolution on the global distribution of BCA, including transport to the Arctic by lows and frontal systems. Using the results of the global scale simulation, we can estimate the total effects of lows and frontal systems generated worldwide. In addition, we can avoid discontinuity effects using the global model. Thus, we conducted a global kilometre-order simulation combined with an aerosol transport module and investigated the impact of the detailed structure of the lows and frontal systems on the transport of BCA to the Arctic.

## Results

### Detailed structure of lows and frontal systems simulated with fine grid resolution

**Figure 1:**
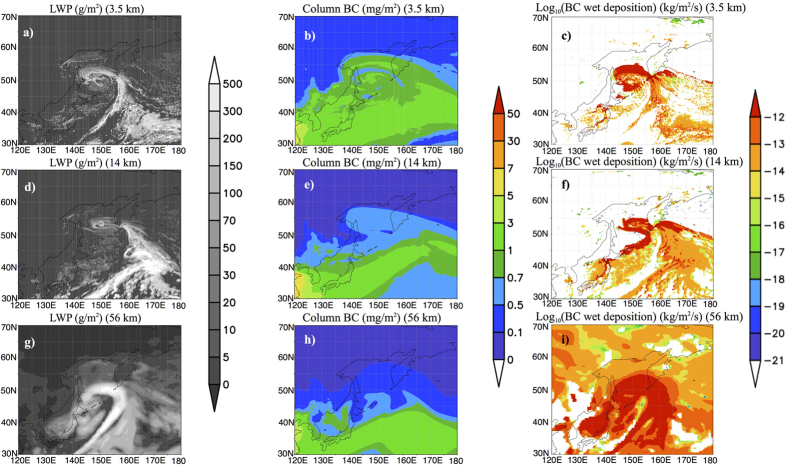
Differences in the structure of a low and frontal system, and black carbon aerosol (BCA) transport for model simulations using different grid resolutions. (**a,d,g**) Example of frontal systems and lows visualised by the vertically accumulated mass of liquid water (liquid water path: LWP) (g m^−2^) at 21UTC on 20111124 over Japan; (**b,e,h**) black carbon aerosol (BCA) transport by the lows and frontal systems shown by the vertically accumulated BCA (column BCA) (mg m^−2^); and (**c,f,i**) wet deposition flux of BCA at the same time. (**a–c**), (**d–f**), and (**g–i**) indicate the results of the simulation at 3.5-, 14-, and 56-km resolution, respectively. The mapping of the figures was created using the Grid Analysis and Display System (GrADS)^51^ version 2.1.a1.

Figure 1 shows the liquid water path, column BCA mass, and wet deposition flux of BCA around a low and the frontal systems accompanying the low. The structures of the low and frontal systems simulated by the model were highly dependent on the grid resolution (Fig. 1a,d,g). The structure of the vortex around (50°N, 150°E) became clear, and the horizontal scale of the cold fronts decreased when the grid resolution was fine. In the results with fine grid resolution, the vortex around the low involved large amounts of BCA that were carried northward by a low (Fig. 1b). This is consistent with the results of a regional model^24^; the filamentary structure of the vortex around lows was clearly reproduced by the fine grid resolution, and BCA transport by the low was larger than was suggested using a coarse grid resolution.

In addition to the components of a frontal system itself, the contrast between cloud and cloud-free areas (*i.e.*, the detailed structure of the clouds) around the systems were well resolved using the fine grid resolution. The detailed structure of clouds reproduced a small-scale contrast between the areas in which wet deposition occurred actively and the areas where wet deposition was inactive (Fig. 1c). The BCAs in the cloud-free area remained in the atmosphere and were readily incorporated into the vortex around the lows. On the other hand, the contrast was obscure and the cloud area was broadly extended in the simulation with a coarse grid resolution (Fig. 1g). This broad area of cloud effectively removed the BCA by wet deposition (Fig. 1i). The ratio of the cloud free area around lows and frontal systems, defined as the ratio of the number of grids whose LWP was smaller than 1 g m^−2^ to the total number of grids with lows and frontal systems (see the ref. 27 for details of the extraction of the systems), increased from 23.30, 42.25, and 51.44% at grid resolutions of 56, 14, and 3.5 km, respectively. As a result of the differences in the cloudy area, the precipitation amount averaged over all lows and frontal systems decreased with the finer grid resolutions to 3.434, 3.389, and 3.178 mm day^−1^, respectively. The small cloud area and low precipitation led to a small number of grids in which wet deposition was active. The amount of aerosol removed by wet deposition decreased at finer grid resolutions.

The difference in the cloud and cloud-free areas at different resolutions is supported by the results of a previous study, which showed that a better representation of convection, clouds, and aerosol–cloud interaction processes of a cloud resolving model resulted in a better performance than that of a model with coarser grid resolution. A previous study underestimated the BCA using a 10-km grid resolution^24^; it was speculated that this underestimation (*O* (10 km)) was derived from the underestimation of background aerosol. However, our results indicated that simulations with a kilometre order (*O* (1 km)) grid resolution can further reduce the underestimation of BCA in the Arctic.

The differences referred to above resulted in a large dependency of the distribution of BCA upon the grid resolution, and transport by the vortex decreased with coarsening grid resolution (Fig. 1b,e,h). These differences in the transport of BCA in lows and frontal systems were common for almost all lows simulated by the model; large amounts of BCA were shown to be transported to the Arctic inside cyclonic circulations around frontal areas.

**Figure 2:**
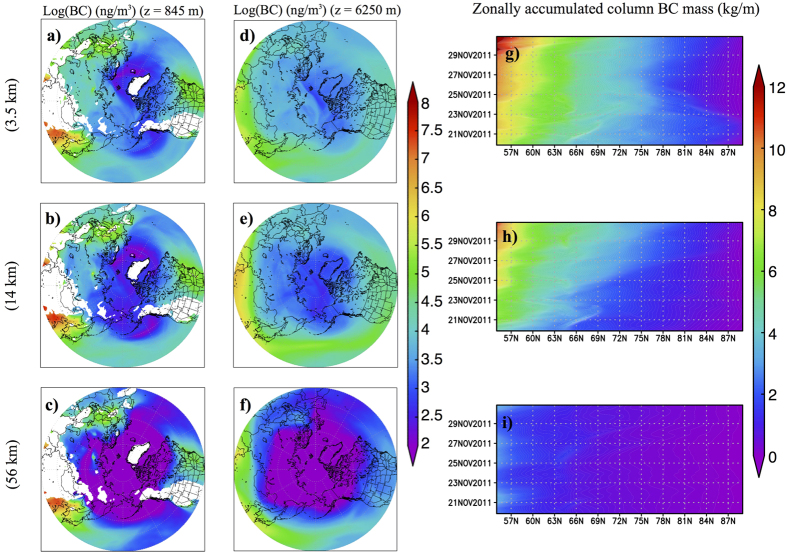
Differences in black carbon aerosol (BCA) transportation to the Arctic for model simulations with three different horizontal resolutions. (**a–f**) Mixing ratio of BCA at (**a–c**) *z* = 845 m (where *z* is the height) and (**d–f**) *z* = 6250 m averaged over the final 10 days of the simulation, and (**g–i**) temporal evolution of zonally accumulated column BCA mass concentration simulated at (**a,d,g**) 3.5-, (**b,e,h**) 14-, and (**c,f,i**) 56-km grid resolutions, respectively. The mapping of the figures was created using the Grid Analysis and Display System (GrADS)^51^ version 2.1.a1.

The dependency of the BCA transport by lows and frontal systems on the grid resolution also led to a difference in BCA mass concentrations over the Arctic (Fig. 2). At low altitudes, with fine grid resolution, BCA from the Eurasian continent (Europe and Russia) was effectively carried to the Arctic (Fig. 2a). In contrast, with coarse grid resolution, transport was low in this region (Fig. 2c). This transport corresponded to low-level transport in the polar front region^21^, which was located further south in winter than in other seasons^22^. Using fine grid resolution, above the boundary layer, a large amount of the BCA was transported to the Arctic regardless of the longitude (Fig. 2d–f). This indicates that BCA emitted from continents was effectively carried up by the convection around lows and frontal systems, and thus reached the Arctic. Due to the dependency of simulated BCA transport on the grid resolution, the BCA concentrations increased in the Arctic with increasing grid resolution. As a result, the ratio of the vertically accumulated mass concentration of BCA (column BCA) in the Arctic to global column BCA mass loading simulated by the 3.5-km resolution model was 4.2-times higher than that simulated by the 56-km resolution model (Table 1).

**Figure 3:**
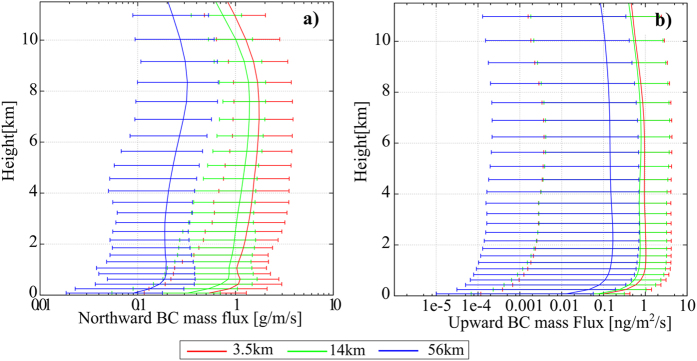
Vertical profile of the northward and upward black carbon aerosol (BCA) mass flux by lows and frontal systems. The vertical profile of the (**a**) zonally accumulated poleward BCA mass flux across 60°N and (**b**) horizontally averaged upward BCA mass flux around lows and frontal systems, simulated with (red) 3.5-km, (green) 14-km, and (blue) 56-km horizontal grid resolution, and averaged during the final 10 days of the simulation. The whiskers of each plot show the range from 5^th^ to 95^th^ percentile. The method used to extract lows and frontal systems were based on the previous study^27^.

The northward mass flux across 60°N and the upward mass flux of BCA by lows and frontal systems (see Supplementary Information for details of the calculations of mass flux) systematically increased at finer grid resolutions over the whole layer (Fig. 3). This indicated that the upward and poleward transport of BCA was enhanced by reproducing the detailed structure of lows and frontal systems. The statistical analysis of the sensitivity of the resolution to BCA transport was also shown quantitatively in the poleward mass flux of BCA across the 60°N latitude line (Table 1). The mass flux gradually increased with a finer grid resolution. In addition to the absolute value of the mass flux, the contribution of lows and frontal systems to the transport of BCA gradually increased with increasing grid resolution. These results all indicate that realistic simulations of the detailed structures of lows and frontal systems increased the simulated amounts of BCA transported from the continents in the Northern Hemisphere by lows and frontal systems.

### Regional variability of BCA transport

**Figure 4:**
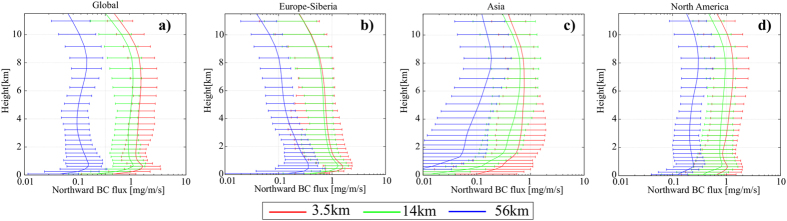
Regional variability of the northward black carbon aerosol (BCA) mass flux. Zonally accumulated vertical profile of the northward BCA mass flux across 60°N over (**a**) the whole globe, (**b**) Europe and Siberia (0°E-120°E), (**c**) Asia (120°E-60°W), and (**d**) North America (60°W-0°E), simulated with (red) 3.5-km, (green) 14-km, and (blue) 56-km horizontal grid resolution, and averaged during the final 10 days of the simulation. The whiskers of each plot show the range from the 5^th^ to 95^th^ percentile.

The total northward BCA mass flux across 60°N including lows and frontal systems, over all vertical layers, systematically increased at finer grid resolutions (Fig. 4a), and the profile had two peaks. The peak in the lower layer corresponded to low-level transport^21^ which was clear in the Europe–Siberia region (0°E-120°E: Fig. 4b). BC emitted from the Siberian region could not be carried into the upper layer due to a strong inversion (called the polar dome^28^), and therefore the dependency was largest in the lower layer in Siberia than in other regions. The peak in the upper layer mainly originated from the flux in the Asian region (120°E-60°W: Fig. 4c) and North America (60°W-0°E: Fig. 4d), which was derived from BCA being elevated by the lows and frontal systems. From these analyses we concluded that BCA transport to the Arctic over the upper (lower) layer was enhanced mainly by the increased transport from the Asian and North America (Siberian) regions generated by reproducing the detailed structure of lows and frontal systems. Such an analysis is one merit of using a global scale model.

### Comparison between the model and observations

**Figure 5:**
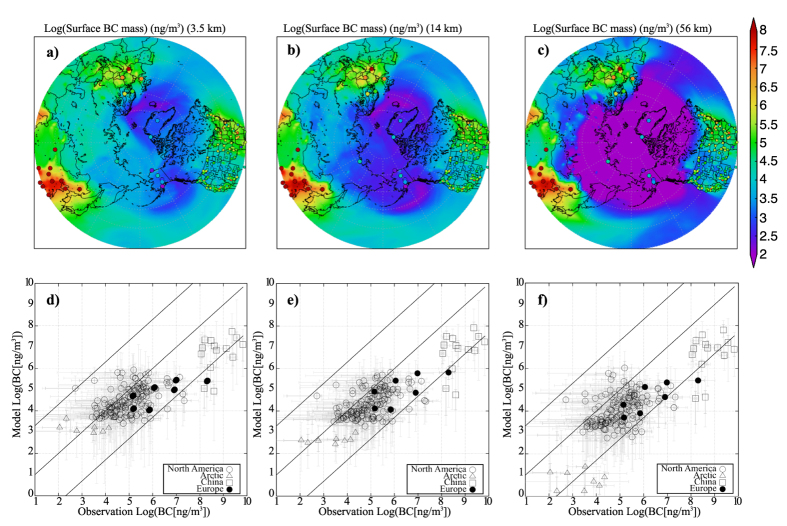
Comparison between surface black carbon aerosol (BCA) simulated by the model and that observed by *in situ* measurements. (**a–c**) Model-derived mass concentrations of surface BCA averaged over the final 10 days of the simulation and (filled circle) those observed by Interagency Monitoring of Protected Visual Environment (IMPROVE^52^), China Atmosphere Watch Network (CAWNET^53^), Canadian Aerosol Baseline Measurement website (CABM), and European Supersites for Atmospheric Aerosol Research (EUSAAR), and (**d–f**) the scatter plot between model-derived BCA mass and observed surface BCA mass. The open circles, triangle, square, and closed circle in (**d–f**) represent results for the observation site in North America, the Arctic, China, and Europe. The thin and thick grey lines in (**d–f**) show the range from minimum to maximum and the 25^th^ to 75^th^ percentile. All data were averaged for November 2011, except for data from CAWNET which were monthly averaged data for November 2006 and 2007. Details of the observation data are provided in Supplementary Information. The mapping of the figures was created using the Grid Analysis and Display System (GrADS)^51^ version 2.1.a1.

A comparison between the model and *in situ* measurements showed that these increased simulated levels of BCA transport contributed to a reduction in the underestimation of BCA in the Arctic (Fig. 5). BCA levels were well reproduced regardless of the resolution, except for Arctic area. In most of the observation sites (except for the Arctic), the BCA level simulated by the model with a fine grid resolution was closer to the observation (Table 2), and the difference in the BCA level among the different resolutions was included in the range of the variability (Fig. 5d–f).

The surface BCA levels in the Arctic differed markedly between the low- and high-resolution models (Table 2), with surface BCA concentrations simulated using the fine grid resolution close to those measured at Arctic observation sites. In addition, the difference between the model with the finest grid resolution (3.5 km) and the observation was smaller than that between the model with 14-km resolution and the observation (Table 2). Hence, simulations using the global-scale kilometre-order model enabled reproduction of lows and frontal systems, and such simulations reduced the underestimation of BCA in the Arctic.

**Figure 6:**
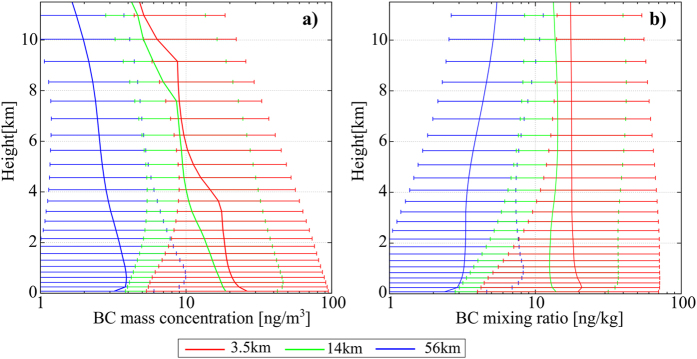
Vertical profile of the black carbon aerosol (BCA). Vertical profile of (**a**) BCA mass concentration and (**b**) mixing ratio of BCA averaged over the Arctic region, simulated with (red) 3.5-km, (green) 14-km, and (blue) 56-km horizontal grid resolution, and averaged during the final 10 days of the simulation. The whiskers of each plot show the range from the 5^th^ to 95^th^ percentile.

As well as a comparison of the surface BCA level, the comparison of vertical profiles of BCA is important because current GCMs generally overestimate BCA in the upper layer^4^. The vertical profile indicated that the BCA level simulated with a fine grid resolution was large in all vertical layers (Fig. 6), which is consistent with the vertical profile of the BCA flux (Fig. 4). Unfortunately, *in situ* measurements of the vertical profile were not conducted on the targeted date (November 2011). In the future, we should conduct experiments targeting the date when the aircraft measurements were conducted to assess the vertical distribution of BCA. However, to understand the model performance, an assessment of the vertical profile through a comparison with the results of previous aircraft measurement is meaningful, even though the measurements were conducted in different seasons.

Based on a previous observational study^5^, the surface BCA level is at a minimum in summer. After the summer, the surface BCA level gradually increases to a maximum in spring. Although surface observation may be representative of the BCA level over the upper layer^29^, BCA over the upper layer displays a similar seasonal cycle to the surface according to several aircraft measurements (Polar Study using Aircraft, Remote Sensing, Surface Measurements, and Models of Climate Chemistry, Aerosols, and Transport: POLARCAT^30^, Arctic Research of the Composition of the Troposphere from Aircraft and Satellites; ARCTAS^31^, Aerosol, Radiation, and Cloud Processes affecting Arctic Climate: ARCPAC^32^, Polar Airborne Measurements and Arctic Regional Climate Model Simulation Project: PAMARCMiP^33^, and High-Performance Instrumented Airborne Platform for Environmental Research Pole-to-Pole Observations: HIPPO^34^,^35^). The BCA level simulated with the coarse resolution was much lower than in the aircraft measurements listed above, but the order of the BCA level simulated by the finest resolution was closer to the aircraft measurement (several to several tenths ng m^−3^) (Fig. 6). However, our model did not show the reduction in the BCA level above the 10-km layer that was observed in spring (ARCTAS campaign) and in fall (HIPPO campaign). From these analyses, we can infer that as in current GCMs, our model also overestimated the BCA level above the boundary layer^36^. To improve the vertical distribution of BCA, a more detailed understanding of the microphysical processes of BCA, as well as a finer grid resolution, are required.

## Discussion

In this study, a global aerosol transport simulation with a kilometre-order horizontal grid resolution was conducted, and the effects of grid resolution on BCA transport to the Arctic were investigated. The results of the simulations indicated that global aerosol transport simulations with fine grid resolution can reduce the underestimation of BCA in the Arctic. An underestimation of BCA still remained even when a fine grid resolution was used. The analyses of the poleward BCA mass flux (Table 1) and the contrast between the cloud and cloud free area showed that the grid convergence on both the contrast and the poleward BCA mass flux were not achieved, even if the finest grid resolution (3.5 km) was used. As well as the analyses, a previous study using a global cloud resolving model proposed that the grid convergence on vertical velocity was not achieved, even if the grid resolution was set to 870 m^37^. A more recent study determined that the trend of the grid convergence on the cloud area was achieved using a grid resolution finer than 1 km^38^. These results indicate that BCA transport to the Arctic area can still be increased by simulations with a finer grid resolution and that a 10-km grid resolution is not sufficient to reproduce the detailed structure of lows and frontal systems and their associated BCA transport. An even finer grid resolution simulation (i.e., kilometre-order or finer) is required.

In addition to refining the grid resolution, sophisticated aerosol microphysics (e.g., aging, wet deposition, and dry deposition), and a detailed emission inventory are also important. Efforts to improve all of these aspects must be undertaken in the future.

High resolution (*i.e.*, kilometre-order) was effective for increasing not only the transport of BCA but also other aerosol species (*e.g.*, sea salt, dust, and sulphate), and thus for advancing our understanding of the effects of aerosols on the global climate system. Our results also indicated that poleward aerosol transport in the Southern Hemisphere may also be underestimated in current-generation GCMs. The aerosol distribution in the Antarctic region should be evaluated through careful comparisons between model simulations and *in situ* measurements.

## Methods

We performed global aerosol transport simulations using the Non-hydrostatic Icosahedral Atmospheric Model (NICAM)^39^,^40^,^41^ coupled with the aerosol module, Spectral Radiation-Transport Model for Aerosol Species (SPRINTARS)^42^. SPRINTARS was developed using a GCM (Model for Interdisciplinary Research on Climate: MIROC) and has been used in international model intercomparison studies (*e.g.*, Hemispheric Transport of Air Pollution; HTAP^43^, and Aerosol Comparison between Observation and Models; AeroCOM^44^). The details of the model are described in Supplementary Information. Simulations were conducted at horizontal grid resolutions of 3.5, 14, and 56 km. The number of vertical layers was 38 for all experiments, and the layer thickness was gradually increased from 80 m near the surface to 5000 m at 38 km (the model top). In this study, we mainly focused on the impact of the horizontal structure of lows and frontal systems on the transport of BCA. The potential impact of the vertical resolution^26^ on the BCA distribution is beyond the scope of this study.

The initial dynamics and sea surface temperatures (SSTs) were derived from the National Center for Environmental Prediction Final Analysis (NCEP-FNL)^45^ data. The sea ice mass was derived from the 1979–1999 monthly climatology obtained from a previous study^46^. To derive the initial condition of the aerosol, we conducted a 2-year simulation using 56-km horizontal resolution based on the initial atmospheric and SST conditions with no initial aerosol. Emission inventories of BCA, organic carbon (OC), and SO_2_ from anthropogenic sources were obtained from the Hemispheric Transport of Air Pollution Phase 2 (HTAP_v2.2)^43^, and the emissions from biomass burning were derived from the Global Fire Emissions Database, version 3 (GFEDv3)^47^,^48^. Terpene and isoprene, which are precursor gases for secondary organic aerosols based on the Global Emissions InitiAtive (GEIA)^49^, were included in the simulation. Prescribed monthly oxidants (OH radicals, ozone, and H_2_O_2_), which were required for chemical reactions involving sulphate, were obtained from the results of the GCM^50^. An emission inventory of SO_2_ from volcanic sources was obtained from a previous study^42^. Numerical integrations were conducted for 14 days from 2011111700UTC to 2011120100UTC with time steps (Δ*t*) of 15, 60, and 240 s for resolutions of 3.5, 14, and 56 km, respectively. Although the length of the time integration (14 days) may not have been sufficient to consider all elements of BCA transport to the Arctic (*e.g.*, as shown in Fig. 5. 1 of a previous study^11^), the time scale of lows and frontal systems ranged from several days to two weeks, and therefore meaningful discussions regarding BCA transport by lows and frontal systems were possible.

## Additional Information

**How to cite this article**: Sato, Y. *et al.* Unrealistically pristine air in the Arctic produced by current global scale models. *Sci. Rep.* **6**, 26561; doi: 10.1038/srep26561 (2016).

## Supplementary Material

Supplementary Information

## Figures and Tables

**Table 1:** Fraction of BCA in the Arctic, mass flux of black carbon aerosol (BCA) to the Arctic, and the contribution of lows and frontal systems (LF) to the mass flux.

Horizontal resolution (km)	56	14	3.5
Ratio of column BCA mass concentration in the Arctic to global column BCA mass concentration (%)	1.0	2.7	4.2
Mass flux of column BCA to the Arctic (kg/s)	8.15	27.66	35.55
Ratio of mass flux by LF to total mass flux (%)	55.95	57.63	58.17

Ratio of column black carbon aerosol (BCA) mass concentration in the Arctic to global column BCA mass concentration; (column BCA accumulated north of 60°N)/(globally accumulated column BCA), and zonally accumulated northward mass flux of column BCA across 60°N averaged during the final 10 days of the simulation. The calculation of mass flux is described in the Supplementary Information. “LF” means lows and frontal systems.

**Table 2:** Comparison between surface observations and the model for several regions.

Horizontal resolution (km)	North America	Europe	China	Arctic
3.5	1.120	1.317	1.324	1.030
14	1.153	1.272	1.314	1.254
56	1.249	1.369	1.340	6.105

Ratio of the surface black carbon aerosol (BCA) concentration from *in situ* measurements to that simulated by the model averaged over sites in North America, Europe, China, and the Arctic.
