# Supplementary material for: Unrealistically pristine air in the Arctic produced by current global scale models
Source: Sci Rep. 2016 May 25;6:26561. doi: 10.1038/srep26561 (PMC4879630; doi:10.1038/srep26561)
Supplement: Supplementary Information [file srep26561-s1.pdf]

Supplementary information of

"Unrealistically pristine air in the Arctic produced by current global scale models"

Yousuke Sato[1], Hiroaki Miura[2], Hisashi Yashiro[1], Daisuke Goto[3], Toshihiko Takemura[4], Hirofumi Tomita[1], and Teruyuki Nakajima[5].

1: RIKEN Advanced Institute for Computational Science, Kobe, Japan

2: Department of Earth and Planetary Science, The University of Tokyo, Tokyo, Japan

3: National Institute of Environmental Studies, Tsukuba, Japan

4: Research Institute for Applied Mechanics, Kyushu University, Fukuoka, Japan

5: Earth Observation Research Center, Japan Aerospace Exploration Agency, Tsukuba, Japan

Corresponding author: Yousuke Sato, RIKEN Advanced Institute for Computational Science, Kobe, Japan (yousuke.sato@riken.jp)

Supplementary Methods

1. Details of the global-scale kilometer-order simulation

Global aerosol transport simulations were conducted using the Non-hydrostatic Icosahedral Atmospheric Model (NICAM)[1] coupled with the aerosol module; Spectral Radiation-Transport Model for Aerosol Species (SPRINTARS)[2]. The model solves fully compressible and non-hydrostatic equations. For spatial discretization, the finite volume method was applied. An icosahedral grid system, and a terrain-following grid system with 38 vertical layers were adopted. The layer thickness was gradually increased from 80 m near the surface to 5000 m at 38 km (the model top). The atmosphere below 2 km was covered by 10 layers. Experiments were conducted with three horizontal grid resolutions: 3.5, 14, and 56 km. A Mellor-Yamada-type turbulence scheme[3], a *k*-distribution radiation scheme (MSTRN-X[4]) , a Louis-type scheme for surface flux[5], MATSIRO land surface scheme[6], and a slab ocean model[7] were also used. Cloud microphysical processes were calculated by a single moment bulk microphysical scheme[8], whose autoconversion and accretion rate were affected by aerosol number concentrations in simulations at 3.5- and 14-km resolution (without cumulus parameterization). Large-scale condensation[9] and cumulus parameterization[10] were used for the simulation at 56-km resolution.

The source and sink processes of BCA were calculated based on SPRINTARS. The source processes included re-emissions from the evaporation of clouds and emissions from the burning of biomass, agricultural waste, and fossil fuels. The sink processes were gravitational settling, scavenging by clouds and raindrops (wet deposition), and dry deposition. Emission inventories of BCA, organic carbon (OC), and SO_2 from anthropogenic sources were provided by the Hemispheric Transport of Air Pollution Phase 2 (HTAP_v2)[11], and the emissions from biomass burning were based on the Global Fire Emissions Database, version 3 (GFEDv3)[12,13]. Terpene and isoprene, which are precursor gases for secondary

organic aerosols based on Global Emissions InitiAtive (GEIA)[14] were included in the simulation. Prescribed monthly oxidants (OH radicals, ozone, and H_2O_2), which are required for chemical reactions involving sulfate, were obtained from the results of the GCM[2] An emission inventory of SO_2 from volcanic sources was obtained from a previous study[2].

The initial dynamics and sea surface temperatures (SSTs) were derived from the National Center for Environmental Prediction Final Analysis (NCEP-FNL)[15] data. Sea ice mass was derived from the 1979–1999 monthly climatology as a previous study[16]. To derive the initial condition of the aerosols, we first conducted a 2-year simulation using a 56-km horizontal resolution based on the initial atmospheric and SST conditions with no initial aerosol.

Numerical integrations were conducted for 14 days from 2011111700UTC to 2011120100UTC with time steps (Δt) of 15, 60, and 240 s for resolutions of 3.5, 14, and 56 km, respectively. The calculations were conducted using 20,480 nodes of the K computer.

2. Method to calculate the mass flux and to extract lows and frontal systems

The upward mass flux of BCA ($F_{BC}|_{up}$) and northward mass flux of BCA ($F_{BC}|_{60N}$) across 60°N at each layer is given as

$$F_{BC}|_{up} = \rho \max(w,0)\ q_{BC}(x,y,z),$$

and

$$F_{BC}|_{60N} = \rho \max(v,0)\ q_{BC}(x,y=60^{o}N,z),$$

where ρ, v, w, q_{BC}, x, and z represent the density, meridional wind, vertical wind, mixing ratio of BCA, longitude, and height, respectively. The vertically integrated mass flux of BCA is given by

$$F_{BC}\big|_{60N} = \sum_x \sum_z \left[\rho \max(v,0) q_{BC}\left(x, y = 60^o N, z\right) \right] \Delta x \Delta z$$,

where *Δx* and *Δz* represent the zonal grid spacing and layer thickness, respectively. The temporal interval of the model output was 3 h.

Lows and frontal systems were extracted to estimate their contribution to BCA transport; they were determined following a previously reported method[17], which was based on column accumulated liquid water mass (liquid water path), a thermal front parameter, and sea level pressure.

3. Surface observation data

The observational data shown in Fig. 5 were obtained from the Interagency Monitoring of Protected Visual Environment (IMPROVE) website (http://vista.cira.colostate.edu/improve/Data/data.htm), China Atmosphere Watch Network (CAWNET)[18], Canadian Aerosol Baseline Measurement website (CABM: http://www.ec.gc.ca/natchem/default.asp?lang=En&n=0AC1992C-1), and European Supersites for Atmospheric Aerosol Research website (EUSAAR: http://www.eusaar.net/).

4. Global distribution of clouds and aerosols simulated by the global aerosol transport model with a kilometer-order resolution

In this section, we demonstrate the validity of our method using 3.5-km simulations. The validity was confirmed through comparisons between the model and satellite observations. Because the aerosol process is closely related to clouds, we confirmed the validity of both aerosols and clouds simulated by the model. The optical thickness of clouds and the effective radii of cloud droplets derived from the radiance data obtained by the

Moderate Resolution Imaging Spectroradiometer (MODIS)-Aqua satellite using the Comprehensive Analysis Program for Cloud Optical Measurement (CAPCOM)[19,20,21] algorithm, and aerosol optical thickness of Level 2 products of MODIS (MOD04_L2; http://modis-atmos.gsfc.nasa.gov/MOD04_L2/index.html) were used as the observational data.

Figure S1 shows the global distribution of retrieved and simulated optical thicknesses of clouds, effective radii of droplets, and optical thicknesses of aerosols. The model successfully reproduced the global distribution of cloud and aerosol properties. The contrast in effective radii between continents and oceans[21,22,23,24] and optically thick cloud over heavily polluted regions[25] (East Asia, North America, and Europe) were well reproduced. The effective radii of droplets in the southern Pacific and southern Indian Ocean were overestimated in the model, due to uncertainties in the diagnostic equation. Except for this overestimation, cloud properties were well reproduced by the model at a 3.5-km grid resolution. Aerosol fields were also well reproduced by the model. These results confirm the validity of the model.

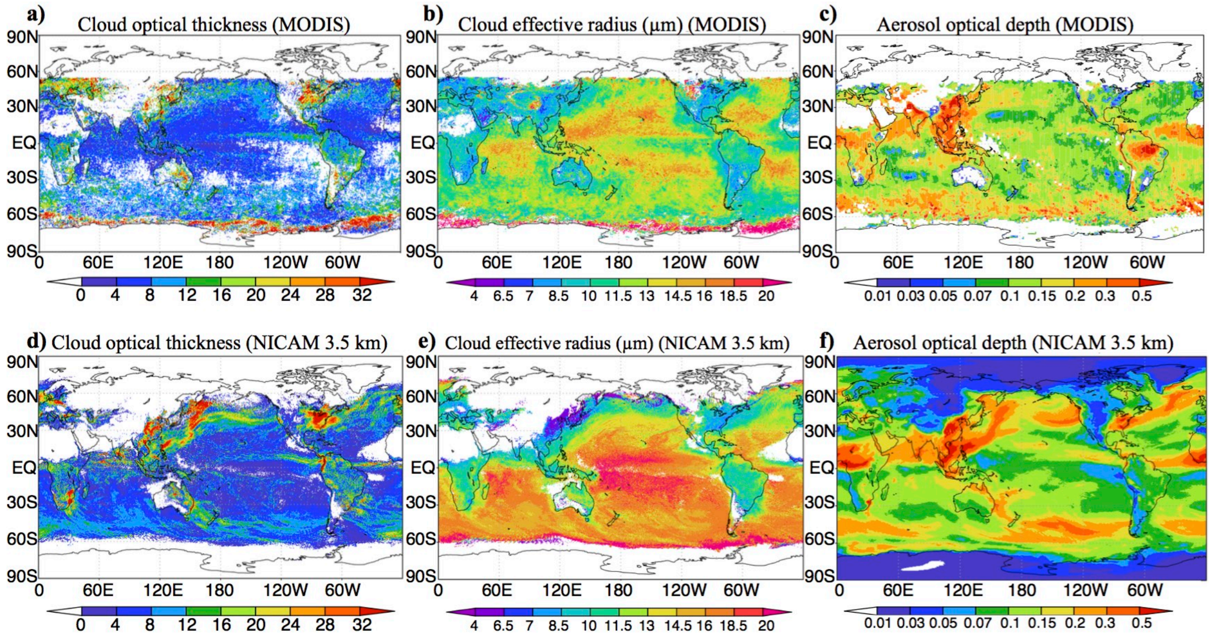

Figure S1: Global distribution of (a, d) cloud optical thicknesses, (b, e) effective radii of cloud droplets at the top of the cloud, (c, f) aerosol optical depths (a–c) retrieved from the Moderate Resolution Imaging Spectroradiometer (MODIS) satellite, and (d–f) simulations of Non-hydrostatic Icosahedral Atmospheric Model – Spectral Radiation-Transport Model for Aerosol Species (NICAM-SPRINTARS) at 3.5-km resolution. The satellite-derived values were averaged for November 2011, and the model-derived values were averaged over the final 10 days of the simulation. The effective radii of cloud droplets by MODIS were retrieved based on a radiance of 3.7 μm, and the effective radii of cloud droplets derived by the model were calculated from an equation used in a previous study[26]. We used the model-derived effective radii of cloud droplets at the top of the cloud because values retrieved from 3.7-μm radiance appeared to be those of the optically thin layer[27,28]. The mapping of the figures was created by using the Grid Analysis and Display System (GrADS)[29] version 2.1.a1.

Supplementary Note

Reference cited in Supplementary Information

1. Satoh, M. *et al.* The Non-hydrostatic Icosahedral Atmospheric Model: description and development. *Prog. Earth Planet. Sci.* **1,** 18 (2014).
2. Takemura, T., Nozawa, T., Emori, S., Nakajima, T. Y. & Nakajima, T. Simulation of climate response to aerosol direct and indirect effects with aerosol transport-radiation model. *J. Geophys. Res.* **110,** D02202 (2005).
3. Nakanishi, M. & Niino, H. An Improved Mellor–Yamada Level-3 Model: Its Numerical Stability and Application to a Regional Prediction of Advection Fog. *Boundary-Layer Meteorol.* **119,** 397–407 (2006).
4. Sekiguchi, M. & Nakajima, T. A k-distribution-based radiation code and its computational optimization for an atmospheric general circulation model. *J. Quant. Spectrosc. Radiat. Transf.* **109,** 2779–2793 (2008).
5. Uno, I., Cai, X.-M., Steyn, D. G. & Emori, S. A simple extension of the Louis method for rough surface layer modelling. *Boundary-Layer Meteorol.* **76,** 395–409 (1995).
6. Takata, K., Emori, S. & Watanabe, T. Development of the minimal advanced treatments of surface interaction and runoff. *Global and Planetary Change* **38,** 209–222 (2003).
7. Maloney, E. D. & Sobel, A. H. Surface fluxes and ocean coupling in the tropical intraseasonal oscillation. *J. Clim.* **17,** 4368–4386 (2004).
8. Tomita, H. New Microphysical Schemes with Five and Six Categories by Diagnostic Generation of Cloud Ice. *J. Meteorol. Soc. Japan* **86A,** 121–142 (2008).

9. Tiedtke, M. Representation of Clouds in Large-Scale Models. *Mon. Weather Rev.* **121,** 3040–3061 (1993).

10. Chikira, M. & Sugiyama, M. Eastward-Propagating Intraseasonal Oscillation Represented by Chikira–Sugiyama Cumulus Parameterization. Part I: Comparison with Observation and Reanalysis. *J. Atmos. Sci.* **70,** 3920–3939 (2013).

11. Janssens-Maenhout, G. *et al.* HTAP_v2.2: a mosaic of regional and global emission grid maps for 2008 and 2010 to study hemispheric transport of air pollution. *Atmos. Chem. Phys.* **15,** 11411–11432 (2015).

12. Randerson, J. T., van der Werf, G. R., Giglio, L., Collatz, G. J. & Kasibhatla, P. S. *Global Fire Emissions Database, Version 3 (GFEDv3.1). Data set. Available on-line [http://daac.ornl.gov/] from Oak Ridge National Laboratory Distributed Active Archive Center*. (2013). doi:10.3334/ORNLDAAC/1191

13. van der Werf, G. R. *et al.* Interannual variability in global biomass burning emissions from 1997 to 2004. *Atmos. Chem. Phys.* **6,** 3423–3441 (2006).

14. Guenther, A. *et al.* A global model of natural volatile organic compound emissions. *J. Geophys. Res.* **100,** 8873 (1995).

15. Kalnay, E. *et al.* The NCEP/NCAR 40-Year Reanalysis Project. *Bull. Am. Meteorol. Soc.* **77,** 437–471 (1996).

16. Kodama, C., Noda, A. T. & Satoh, M. An assessment of the cloud signals simulated by NICAM using ISCCP, CALIPSO, and CloudSat satellite simulators. *J. Geophys. Res. Atmos.* **117,** (2012).

17. Miyamoto, Y. *et al.* Does convection vary in different cloud disturbances? *Atmos. Sci. Lett.* **16,** 305–309 (2015).

18. Zhang, X. Y. *et al.* Atmospheric aerosol compositions in China: Spatial/temporal variability, chemical signature, regional haze distribution and comparisons with global aerosols. *Atmos. Chem. Phys.* **12,** 779–799 (2012).

19. Nakajima, T. & King, M. D. Determination of the Optical Thickness and Effective Particle Radius of Clouds from Reflected Solar Radiation Measurements. Part I: Theory. *J. Atmos. Sci.* **47,** 1878–1893 (1990).

20. Nakajima, T. Y. & Nakajma, T. Wide-Area Determination of Cloud Microphysical Properties from NOAA AVHRR Measurements for FIRE and ASTEX Regions. *J. Atmos. Sci.* **52,** 4043–4059 (1995).

21. Kawamoto, K., Nakajima, T. & Nakajima, T. Y. A Global Determination of Cloud Microphysics with AVHRR Remote Sensing. *J. Clim.* **14,** 2054–2068 (2001).

22. Squires, P. The Microstructure and Colloidal Stability of Warm Clouds. *Tellus* **10,** 256–261 (1958).

23. Han, Q., Rossow, W. B. & Lacis, A. A. Near-Global Survey of Effective Droplet Radii in Liquid Water Clouds Using ISCCP Data. *J. Clim.* **7,** 465–497 (1994).

24. Nakajima, T. Y., Masunaga, H. & Nakajima, T. Near-Global Scale Retrieval of the Optical and Microphysical Properties of Clouds from Midori-II GLI and AMSR Data. *J. Remote Sens. Soc. Japan* **29,** 29–39 (2009).

25. Twomey, S. The Influence of Pollution on the Shortwave Albedo of Clouds. *Journal of the Atmospheric Sciences* **34,** 1149–1152 (1977).

26. Suzuki, K. *et al.* Global cloud-system-resolving simulation of aerosol effect on warm clouds. *Geophys. Res. Lett.* **35,** L19817 (2008).

27. Platnick, S. Vertical photon transport in cloud remote sensing problems. *J. Geophys. Res.* **105,** 22919–22935 (2000).

28. Nakajima, T. Y., Suzuki, K. & Stephens, G. L. Droplet Growth in Warm Water Clouds Observed by the A-Train. Part I: Sensitivity Analysis of the MODIS-Derived Cloud Droplet Sizes. *J. Atmos. Sci.* **67,** 1884–1896 (2010).

29. Institute for Global Environment and Society (IGES). Grid Analysis and Display System (GrADS). (1989). at <http://www.iges.org/grads/grads.html>
